# Increased Toxicity of *Karenia brevis* during Phosphate Limited Growth: Ecological and Evolutionary Implications

**DOI:** 10.1371/journal.pone.0058545

**Published:** 2013-03-12

**Authors:** Donnie Ransom Hardison, William G. Sunda, Damian Shea, Richard Wayne Litaker

**Affiliations:** 1 National Oceanic and Atmospheric Administration, National Ocean Service, Center for Coastal Fisheries and Habitat Research, Beaufort, North Carolina, United States of America; 2 Department of Biology, North Carolina State University, Raleigh, North Carolina, United States of America; University of Connecticut, United States of America

## Abstract

*Karenia brevis* is the dominant toxic red tide algal species in the Gulf of Mexico. It produces potent neurotoxins (brevetoxins [PbTxs]), which negatively impact human and animal health, local economies, and ecosystem function. Field measurements have shown that cellular brevetoxin contents vary from 1–68 pg/cell but the source of this variability is uncertain. Increases in cellular toxicity caused by nutrient-limitation and inter-strain differences have been observed in many algal species. This study examined the effect of P-limitation of growth rate on cellular toxin concentrations in five *Karenia brevis* strains from different geographic locations. Phosphorous was selected because of evidence for regional P-limitation of algal growth in the Gulf of Mexico. Depending on the isolate, P-limited cells had 2.3- to 7.3-fold higher PbTx per cell than P-replete cells. The percent of cellular carbon associated with brevetoxins (%C-PbTx) was ∼ 0.7 to 2.1% in P-replete cells, but increased to 1.6–5% under P-limitation. Because PbTxs are potent anti-grazing compounds, this increased investment in PbTxs should enhance cellular survival during periods of nutrient-limited growth. The %C-PbTx was inversely related to the specific growth rate in both the nutrient-replete and P-limited cultures of all strains. This inverse relationship is consistent with an evolutionary tradeoff between carbon investment in PbTxs and other grazing defenses, and C investment in growth and reproduction. In aquatic environments where nutrient supply and grazing pressure often vary on different temporal and spatial scales, this tradeoff would be selectively advantageous as it would result in increased net population growth rates. The variation in PbTx/cell values observed in this study can account for the range of values observed in the field, including the highest values, which are not observed under N-limitation. These results suggest P-limitation is an important factor regulating cellular toxicity and adverse impacts during at least some *K. brevis* blooms.

## Introduction

Blooms of the toxic dinoflagellate *Karenia brevis* produce a suite of structurally related neurotoxins, brevetoxins (PbTxs), which adversely affect both human and ecosystem health. These toxins bind to voltage-gated sodium channels which results in persistent activation of neuronal, skeletal muscle and cardiac cells [1]. Shellfish feeding on *K. brevis* accumulate PbTxs, which can lead to neurotoxic shellfish poisoning (NSP). NSP symptoms in humans include gastrointestinal problems, nausea, vomiting, dizziness, slurred speech, numbness of lips, mouth and tongue, and respiratory distress [2],[3]. Exposure is enhanced when *K brevis* cells are disrupted by breaking waves and form toxic aerosols [4]. Onshore winds transport these aerosols over beaches and nearshore communities, causing respiratory related illnesses [4]–[6]. PbTxs produced by *K. brevis* also cause the intoxication and death of marine organisms including copepods, fish, bottlenose dolphins and manatees [7]–[10]. The adverse environmental and health effects of *K. brevis* blooms, in conjunction with the associated negative publicity, result in significant economic losses in local communities that depend on tourism and recreational fishing [11]–[13]. The most heavily impacted region is the Gulf of Mexico, especially the west coast of Florida, which experiences toxic *K. brevis* blooms on a nearly annual basis [14].

Several theories have been proposed concerning the factors that control the development and persistence of toxic *K. brevis* blooms along the west coast of Florida. It is generally accepted that the early phase of a bloom is initiated by northerly winds which promote upwelling events that transport nutrients towards the surface and advect *Karenia* cells towards shore where they concentrate along frontal boundaries [15]–[17]. In addition, nutrient assessments of cells from the field indicate that the growth of *K. brevis* blooms is limited by available nitrogen (N) or phosphorus (P), suggesting that these nutrients play an important role in the development and maintenance of blooms [18], [19]. What is less clear are the sources of N and P utilized by these blooms. Upwelling of subsurface nutrients, land runoff, N_2_-fixation, drainage from phosphate mines and atmospheric deposition have all been proposed as important nutrient sources [15], [18], [19]. In reality, some combination of these different sources likely controls the nutrient supply needed to support intense blooms.

Recent laboratory experiments indicate that N-limitation directly affects not only the growth potential of blooms, but also the toxicity of *K. brevis* cells [20]. Intracellular PbTx concentrations (fg/µm^3^) increased by up to 2.5-fold during N-limited growth in laboratory cultures. In the field, this would translate into the potential for a significantly higher PbTx flux into the food web and increased exposure of affected organisms during periods of N-limitation of algal growth [21], [22]. Whether P-limitation causes a similar increase in cellular toxicity is unknown and was the focus of this study. Understanding the degree to which P-limitation regulates the toxicity of *K. brevis* cells is important given the apparent long-term shift from N- toward P-limitation in the Gulf of Mexico and other regions of the subtropical North Atlantic Ocean basin [23]-[25]. P-limitation on the West Florida Shelf is controversial given the presence of phosphate containing soils on the northern and central Florida peninsula. The mining and drainage of these deposits may result in unknown inputs of phosphorus to local rivers and estuaries, but the amount that moves offshore appears to be relatively low [18]. Consistent with this observation, researchers have reported evidence for P-limitation of *Karenia brevis* blooms on the West Florida Shelf [19], [26].

Typically, toxin content per cell increases dramatically when algal growth becomes P-limited. For example, hemolytic activity per cell in the prymnesiophytes *Prymnesium parvum* and *Chrysochromulina polylepis* increases by up to 10-fold under P-limitation [27], [28]. Similarly, P-limitation increases the cellular content of the neurotoxin domoic acid in the diatom *Pseudo-nitzschia multiseries* [29], [30], and increases the toxicity of the dinoflagellate *Karlodinium veneficum* by increasing production of the more toxic congener karlotoxin-1 [31], [32]. It also increases cellular levels of the potent phosphatase inhibitor nodularin in the cynaobacterium *Nodularia spumigena* [33]. Likewise, isolates of the dinoflagellates *Alexandrium tamarens* and *A. minutum*, which failed to produce significant levels of paralytic shellfish poisoning (PSP) toxins under N-limitation, increased their cellular toxin contents under P-limitation [34]. In cases where cellular toxins increase under both N- and P-limitation, the increase in toxin per cell is often higher in P-limited cells [34].

A collection of field measurements made in the Gulf of Mexico indicated that PbTx contents of *K. brevis* cells vary between 1 and 68 pg/cell (Table 1). In a previous study we found that N-limitation could only account for toxin values in the range of 7–25 pg/cell [20]. Observed patterns from the studies cited above suggests that P-limitation, rather than N-limitation might account for the upper range in PbTx contents per cell observed in the field. In this study we investigated the effect of P-limitation on cellular growth rate, and cellular content of chlorophyll *a* (chl *a*), carbon (C), phosphorus (P), nitrogen (N) and PbTxs in laboratory cultures of *K. brevis*.

**Table 1:** Mean values, standard deviations (SD), and ranges (min and max) for total brevetoxins per cell (pg/cell) from various culture and field studies.

	Brevetoxin content (pg/cell)				
Origin of data	Mean	SD	Min	Max	n
Culture strains [88]	11	4	6	16	6
Surf zone [89]	26	20	2	61	15
Surf zone [5]	18	3	12	23	8
Surface samples [67]	24	11	8	47	20
Bottom samples	6	5	3	16	26
Surface and bottom samples [47]	16	11	1	68	118

Previous work showed that increases in cellular PbTx concentrations under N-limitation were consistent with the carbon:nutrient balance (CNB) hypothesis [20]. This hypothesis predicts that nutrient-limited growth is accompanied by a diversion of fixed carbon into increased levels of defensive compounds or structures [35]–[37]. This C diversion into defensive compounds has a dual advantage. It affords greater protection against grazers and pathogens to compensate for the reduced rates of growth and reproduction. It also provides the cell with a means disposing of unneeded fixed carbon during the onset of nutrient limitation of growth, thereby protecting the photosynthetic electron transport chain from over reduction and attendant oxidative stress [38]–[40]. The CNB hypothesis predicts a similar increase in cellular PbTx concentrations should also occur under P-limitation.

Our experiments utilized five different strains of *K. brevis* from varying geographic locations: four from different locations on the west coast of Florida and one from Texas (Table 2). The strains were used to determine how the brevetoxin content of genetically distinct isolates responded to P-limited growth. Because cell size varies with nutrient limitation, PbTx was normalized on a per cell basis, a per biovolume basis, and cell carbon basis. Normalization of brevetoxins to cell volume or cell carbon made it possible to separate physiological changes in toxin per unit cell biomass from seeming changes in toxicity due solely to changes in cell size.

**Table 2:** Isolation date and location of *Karenia brevis* strains.

Strain	Isolation date	Location
CCMP 2228	2001	Mote Marine Laboratory’s New Pass Dock, Sarasota, Florida, USA
CCMP 2229	2001	Offshore of Manasota Key, Florida, USA
CCMP 2820	2007	New Pass Bridge, Sarasota, Florida, USA
CCFWC268 (Wilson)	1953	John’s Pass, Florida, USA
SP2	1999	Offshore of South Padre Island, Texas, USA

## Materials and Methods

### 2.1 Strains and culture conditions

The effect of P-limitation on the cell content of individual brevetoxin congeners and total PbTx concentrations was studied in five *Karenia brevis* strains with different specific growth rates, cell sizes, and P:C ratios (Tables 2 & 3). The Florida strains CCMP 2228, CCMP 2229, and CCMP 2820 were obtained from the Provasoli-Guillard National Center for Marine Algae and Microbiota (West Boothbay Harbor, ME, USA); the Florida Wilson strain (CCFWC268) was acquired from the Fish and Wildlife Research Institute (St. Petersburg, FL, USA); and the Texas strain SP2 was obtained from Dr. Ed Buskey of University of Texas Marine Science Institute (Port Aransas, TX, USA).

**Table 3:** Average values ± standard deviations for growth rate (d^−1^), volume per cell (µm^3^/cell), chlorophyll *a* (fg/µm^3^), total brevetoxins (fg/µm^3^, pg/cell, and brevetoxin carbon as a percent of cellular C [%C-PbTx]), PbTx production rate ([mmol PbTx C/mol C_cell_]/d), cellular P:C ratio (mmol/mol), cellular N:C ratio (mmol/mol) and cellular C normalized to biovolume (mol C/L_cell_) for P-replete and P-limited experimental cultures of *Karenia brevis*.

Phosphate Replete		CCMP 2228	Wilson	SP2	CCMP 2820	CCMP 2229
Growth rate	(d^−1^)	0.473±0.002	0.327±0.006	0.259±0.004	0.417±0.002	0.467±0.002
Volume per cell	(µm^3^/cell)	3650±249	5668±416	5245±126	3826±249	4711±272
Chlorophyll *a*	(fg/µm^3^)	1.28±0.09	1.33±0.02	1.14±0.17	1.57±0.15	1.60±0.12
Total brevetoxin	(fg/µm^3^)	2.51±0.63	2.68±0.61	2.34±0.33	1.43±0.35	1.70±0.51
	(pg/cell)	8.90±2.65	15.2±3.8	12.1±1.6	5.45±1.26	7.83±2.41
	(%C-PbTx)	1.43±0.47	2.1±0.54	1.53±0.35	1.05±0.22	0.75±0.29
PbTx production rate	([mmol PbTx C/mol C_cell_]/d)	6.76±2.22	6.87±1.77	3.96±0.91	4.38±0.92	3.50±1.35
Cellular P:C	(mmol/mol)	7.40±1.73	20.0±7.5	12.9±0.8	12.1±2.1	6.86±0.89
Cellular N:C	(mmol/mol)	125±5	100±7	133±8	137±1	95±10
Cellular C/biovolume	(mol C/L_cell_)	10.9±1.43	7.48±1.42	7.43±1.41	7.67±0.74	11.6±1.39
Phosphate Limited						
Growth rate	(d^−1^)	0.099±0.004	0.090±0.017	0.100±0.006	0.135±0.007	0.152±0.006
Volume per cell	(µm^3^/cell)	5993±316	7687±1358	5551±438	4039±478	5571±395
Chlorophyll *a*	(fg/µm^3^)	0.69±0.22	0.94±0.04	0.58±0.11	0.80±0.18	0.71±0.17
Total brevetoxin	(fg/µm^3^)	7.26±2.37	4.04±0.67	4.90±0.75	3.06±0.77	4.89±1.39
	(pg/cell)	42.7±16.2	29.8±8.3	26.9±4.7	12.7±2.0	26.7±9.5
	(%C-PbTx)	5.30±1.56	2.80±0.99	4.47±0.42	1.64±0.69	2.91±0.62
PbTx production rate	([mmol PbTx C/mol C_cell_]/d)	5.25±1.56	2.52±1.01	4.47±0.50	2.21±0.94	4.42±0.96
Cellular P:C	(mmol/mol)	3.46±1.82	5.21±1.21	4.49±2.20	6.23±1.97	3.76±1.12
Cellular N:C	(mmol/mol)	96±11	99± 9	120±13	95±10	91±7
Cellular C/biovolume	(mol C/L_cell_)	9.92±2.73	8.35±1.75	6.36±1.52	11.3±1.80	10.6±3.11

Cells were cultured in a Percival Scientific model I-36VLX incubator maintained at a constant temperature of 23^o^C and on a 14h:10h daily light:dark cycle to simulate summer light conditions. Photosynthetically active radiation (PAR) was provided at an intensity of 120 µmol quanta m^−2^ s^−1^ via vertically mounted fluorescent Duro-test Vita-lites. PAR intensity was measured with a Biospherical Instruments Inc. QSL-100 4π wand type light meter.

Media consisted of 1.0 L of 0.2 µm filtered Gulf Stream seawater (salinity 36) held in 2.5-L polycarbonate bottles. The media contained added vitamins (0.074 nM vitamin B_12_, 0.4 nM biotin, and 60 nM thiamine), 10 nM Na_2_SeO_3_, and an EDTA-trace metal buffer system [41] (100 µM EDTA, 1 µM FeEDTA, 50 nM MnCl_2_, 40 nM CuCl_2_, 100 nM ZnSO_4_, and 40 nM CoCl_2_). Nutrient-replete culture media contained 64 µM NaNO_3_ and 4 µM NaH_2_PO_4_ (N:P  =  16:1, the Redfield ratio) while P-limited media contained 64 µM NaNO_3_ and 0.5 µM NaH_2_PO_4_ (N:P  =  128:1). Media were sterilized by microwave treatment [42]. Culture pH was measured initially and throughout each experiment with a Thermo Orion 3 Star pH meter equipped with a Ross ultra-combination pH electrode to ensure no carbon dioxide limitation occurred. Culture pH ranged from 8.10 to 8.35 for P-limited cultures and 8.10 to 8.30 for nutrient-replete cultures. *Karenia brevis* cells in our experiments were grown in semi-continuous 2.5-L batch cultures. Experimental cells in both high and low phosphate media were inoculated from acclimated nutrient-sufficient cultures that had been growing exponentially for several weeks at their maximum rates. The P-limited cultures were grown in low phosphate medium until their growth became P-limited. They were then diluted with fresh low-phosphate medium every 2 to 3 days at an average rate of 0.1 d^−1^ to obtain continuous P-limited growth. Nutrient sufficient cultures growing at their maximum rates were diluted sequentially with high phosphate medium well before they reached their maximum cell density to ensure no nutrient limitation of growth rate occurred. Specific growth rates and associated standard errors were calculated by linear regressions of the natural log of biovolume (µL_cells_/L_media_) versus time after correcting for serial culture dilutions [39].

### 2.2 Cell concentrations, mean volume, growth rate, chlorophyll *a*, nutrient element stoichiometry, and brevetoxins

**Figure 1:**
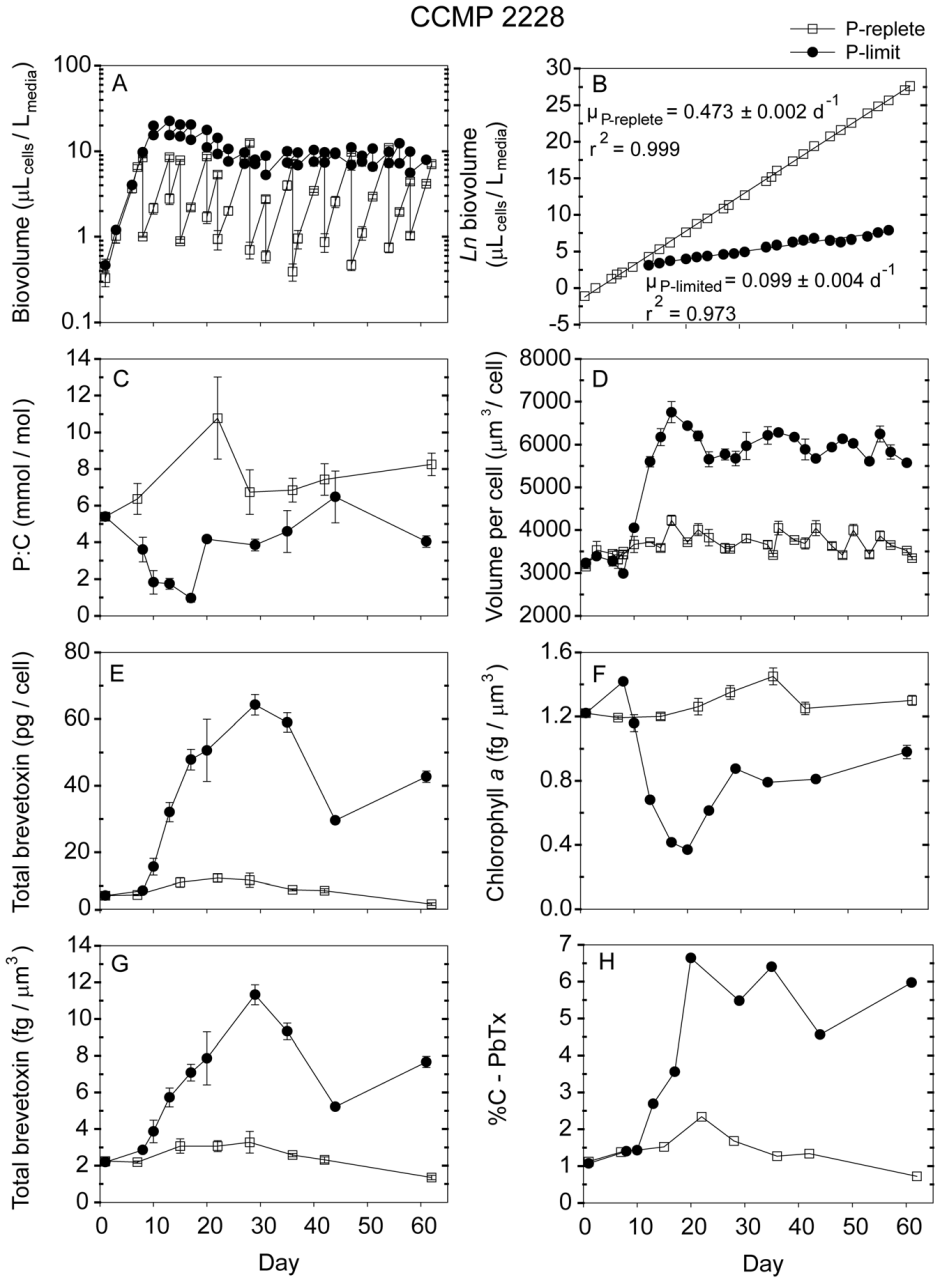
Time-dependent results for semi-continuous batch cultures of *Karenia brevis* strain CCMP 2228 grown at nutrient-replete rates in high-P media (open squares) and grown into P-limitation in low-P media (filled circles). (A) Biovolume (µL_cells_/L_media_) plotted on a log scale vs. time, (B) Natural logarithm (ln) biovolume vs. time, correcting for culture dilution [39], (C) Cellular phosphorus to carbon molar ratios (mmol/mol), (D) Mean volume per cell (µm^3^/cell), (E) Total brevetoxins per cell (pg/cell), (F) Chlorophyll *a* normalized to cell volume (fg/µm^3^), (G) Total brevetoxins normalized to cell volume (fg/µm^3^), (H) Percent of cellular carbon associated with brevetoxins (%C-PbTx). Error bars represent the standard deviation of triplicate measurements, except those normalized to carbon, where the cellular C was measured only in duplicate.

**Figure 2:**
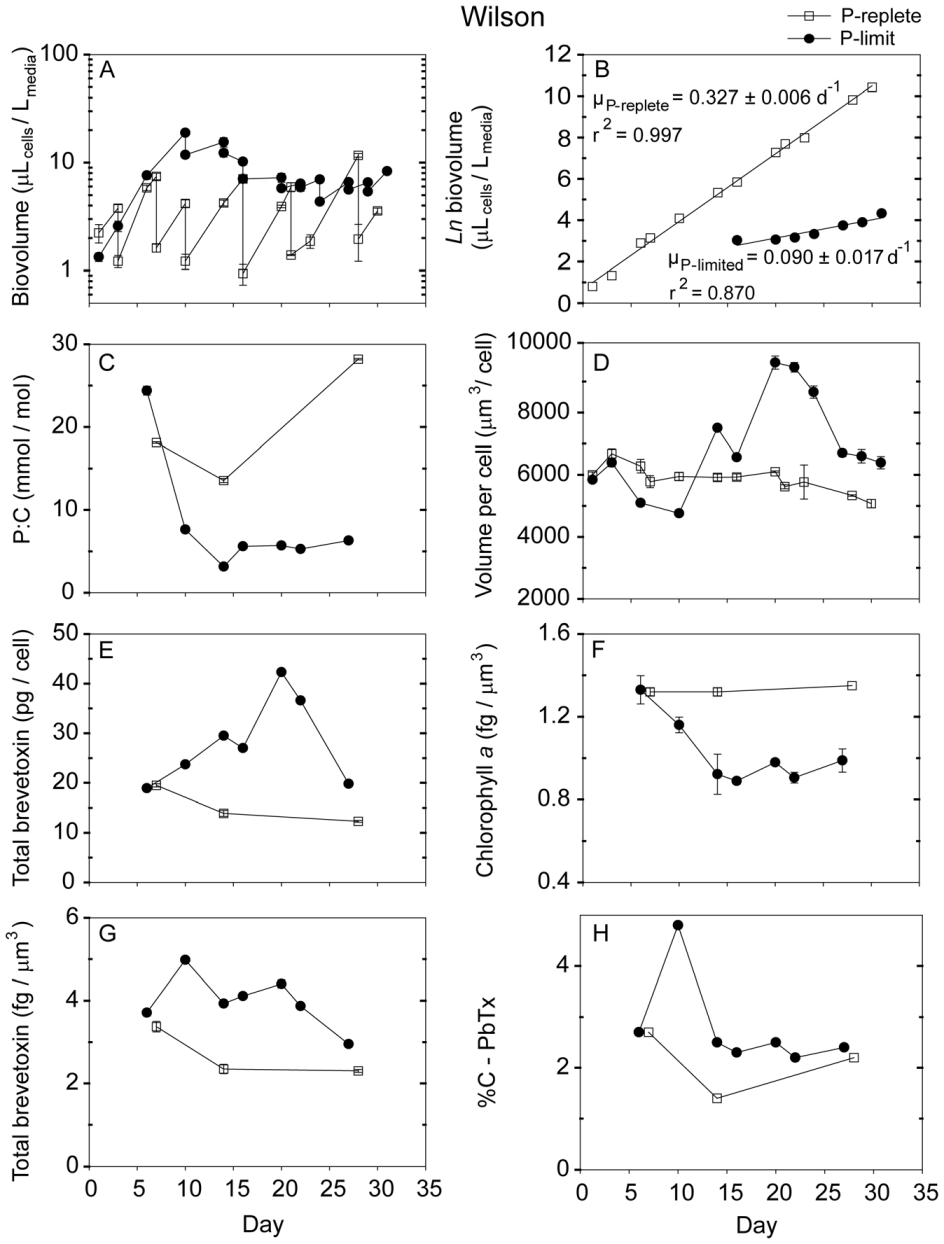
Time-dependent results from semi-continuous batch cultures of *Karenia brevis* strain Wilson grown at nutrient-replete rates in high-P media (open squares) and grown into P-limitation in low-P media (filled circles). (A) Biovolume (µL_cells_/L_media_) plotted on a log scale vs. time, (B) Natural logarithm (ln) biovolume vs. time, correcting for culture dilution [39], (C) Cellular P:C ratios (mmol/mol), (D) Mean volume per cell (µm^3^/cell), (E) Total brevetoxins per cell (pg/cell), (F) Chlorophyll *a* normalized to cell volume (fg/µm^3^), (G) Total brevetoxins normalized to cell volume (fg/µm^3^), (H) Percent of cellular carbon associated with brevetoxins (%C-PbTx). Error bars represent the standard deviation of triplicate measurements, except those normalized to carbon, where the cellular C was measured only in duplicate.

**Figure 3:**
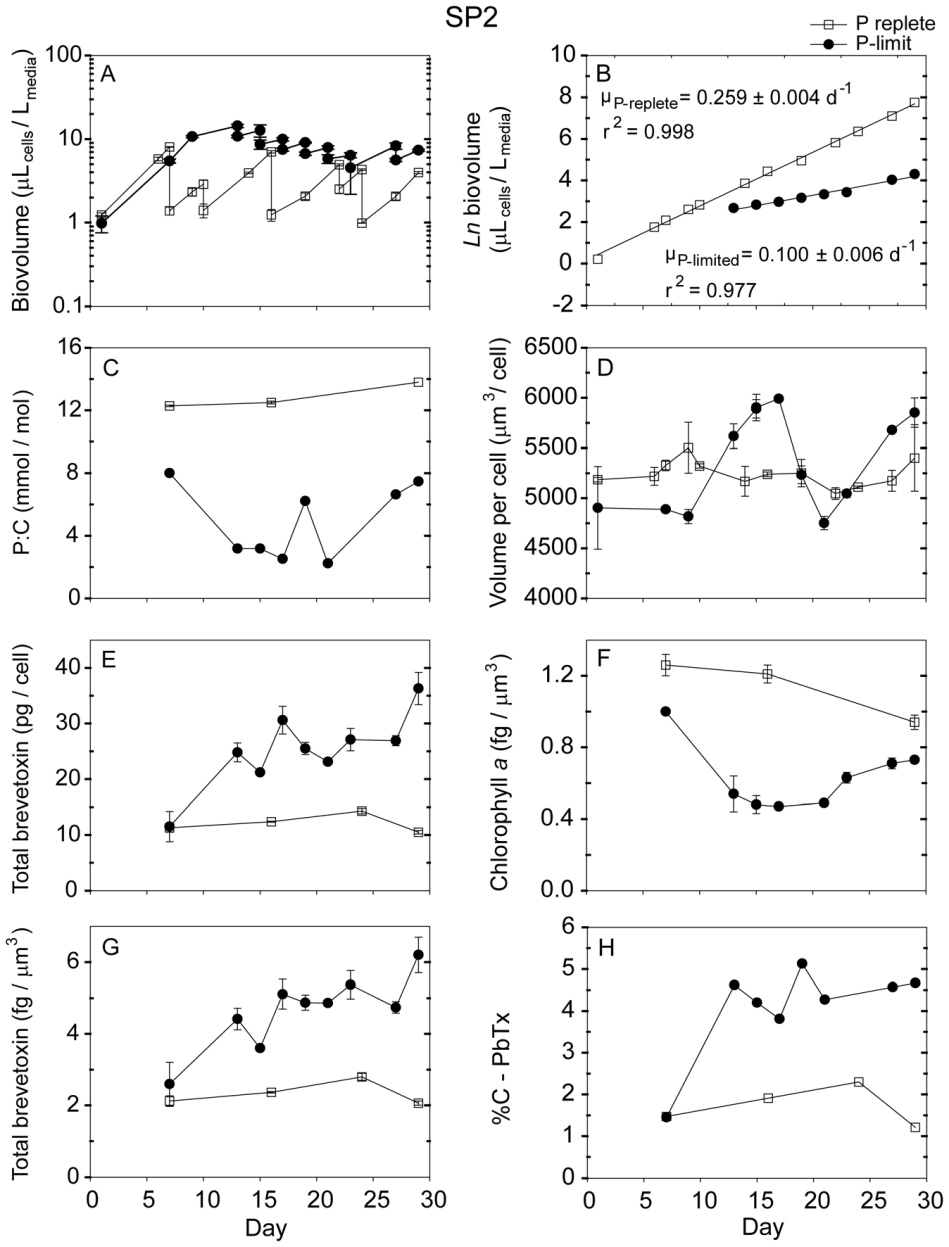
Time-dependent results from semi-continuous batch cultures of *Karenia brevis* strain SP2 grown at nutrient-replete rates in high-P media (open squares) and grown into P-limitation in low-P media (filled circles). (A) Biovolume (µL_cells_/L_media_) plotted on a log scale vs. time, (B) Natural logarithm (ln) biovolume vs. time, correcting for culture dilution [39], (C) Cellular P:C ratios (mmol/mol), (D) Mean volume per cell (µm^3^/cell), (E) Total brevetoxins per cell (pg/cell), (F) Chlorophyll *a* normalized to cell volume (fg/µm^3^), (G) Total brevetoxins normalized to cell volume (fg/µm^3^), (H) Percent of cellular carbon associated with brevetoxins (%C-PbTx). Error bars represent the standard deviation of triplicate measurements, except those normalized to carbon, where the cellular C was measured only in duplicate.

**Figure 4:**
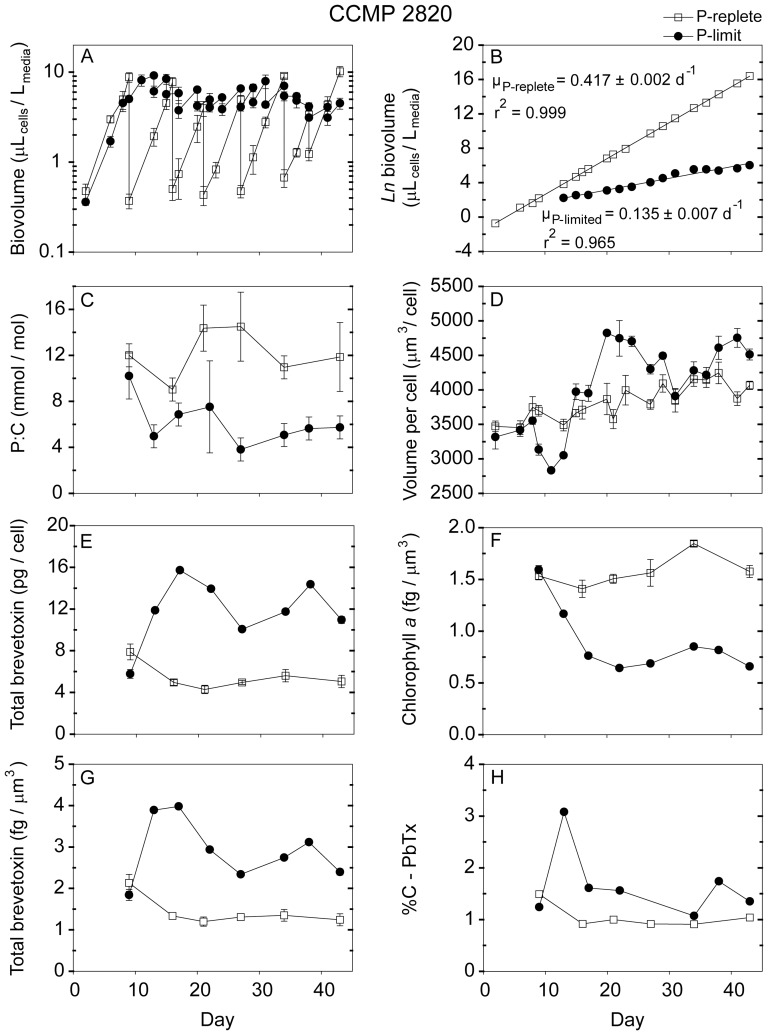
Time dependent results from semi-continuous batch cultures a *Karenia brevis* strain CCMP 2820 grown at nutrient-replete rates in high-P media (open squares) and grown into P-limitation in low-P media (filled circles). (A) Biovolume (µL_cells_/L_media_) plotted on a log scale vs. time, (B) Natural logarithm (ln) biovolume vs. time, correcting for culture dilution [39], (C) Cellular P:C ratios (mmol/mol), (D) Mean volume per cell (µm^3^/cell), (E) Total brevetoxins per cell (pg/cell), (F) Chlorophyll *a* normalized to cell volume (fg/µm^3^), (G) Total brevetoxins normalized to cell volume (fg/µm^3^), (H) Percent of cellular carbon associated with brevetoxins (%C-PbTx). Error bars represent the standard deviation of triplicate measurements, except those normalized to carbon, where the cellular C was measured only in duplicate.

**Figure 5:**
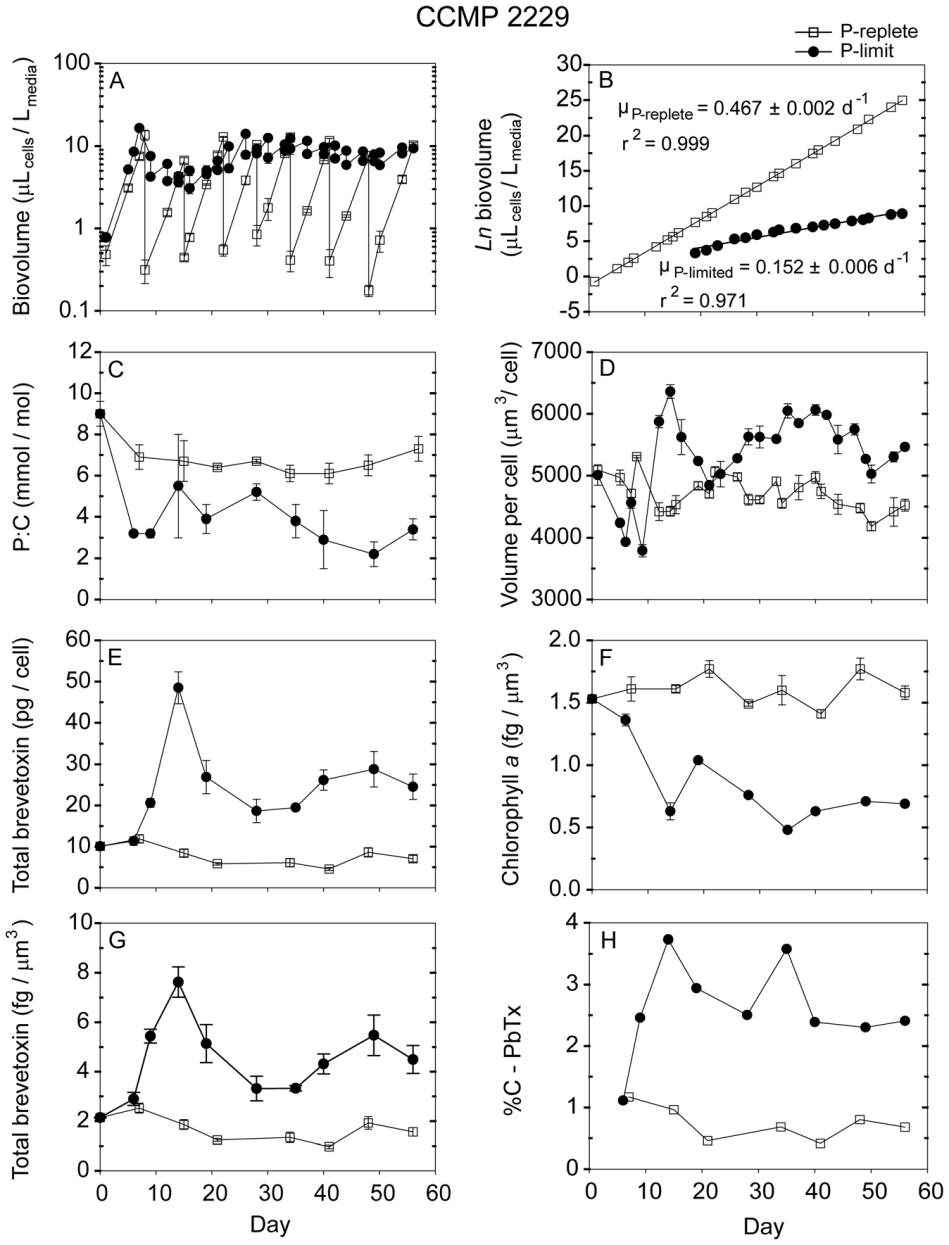
Time dependent results from semi-continuous batch cultures a *Karenia brevis* strain CCMP 2229 grown at nutrient-replete rates in high-P media (open squares) and grown into P-limitation in low-P media (filled circles). Biovolume (µL_cells_/L_media_) plotted on a log scale vs. time, (B) Natural logarithm (ln) biovolume vs. time, correcting for culture dilution [39], (C) Cellular P:C ratios (mmol/mol), (D) Mean volume per cell (µm^3^/cell), (E) Total brevetoxins per cell (pg/cell), (F) Chlorophyll *a* normalized to cell volume (fg/µm^3^), (G) Total brevetoxins normalized to cell volume (fg/µm^3^), (H) Percent of cellular carbon associated with brevetoxins (%C-PbTx). Error bars represent the standard deviation of triplicate measurements, except those normalized to carbon, where the cellular C was measured only in duplicate.

In the middle of the light period (midday) culture aliquots were taken for measurement of cell concentrations and mean volume per cell every 2–3 days for both P-limited and control cultures. Midday samples were also taken for chlorophyll *a* (chl *a*), brevetoxins, and cellular carbon, nitrogen, and phosphorus every 2–4 days, with less frequent sampling near end of some experiments (see Figs. 1, 2, 3, 4, 5). All analyses were conducted in triplicate except for cellular carbon and nitrogen which were measured in duplicate subsamples due to culture volume limitations. Results were expressed as mean values ± standard deviation unless otherwise noted. Cell concentrations and mean volume per cell were measured with a Beckman Coulter Inc. Multisizer 3 electronic particle counter (0.5-mL sample volume) equipped with a 100-µm aperture. Cell growth curves were constructed as semi-log plots of total biovolume (µL_cells_/L_media_) versus time in days. Total biovolume (µL_cells_/L_media_) was calculated by multiplying the cellular concentration (cells/L) by the mean volume per cell (µL/cell). Specific growth rates were computed from linear regressions of the natural log of total cell biovolume versus time after correcting for culture dilution [39].

Chl *a* was measured by filtering cells onto 25-mm GF/F filters and extracting the cells with a 90:10 acetone:water mixture. The fluorescence of the extracted chl *a* was measured with a Turner Design 10-AU fluorometer [43]. Cellular carbon (C) and nitrogen (N) were determined by gentle filtration of cells onto precombusted 13-mm GF/F glass fiber filters [20] followed by fuming with HCl overnight to remove inorganic carbon [44]. These filter samples were then analyzed for cellular N and C with an EAS 4010 Costech elemental analyzer. Cellular phosphorus (P) samples were prepared by gently filtering culture samples onto precombusted 25 mm GF/F filters and analyzing the collected cells for particulate P [45].

Brevetoxins were extracted using liquid/liquid separations with ethyl acetate. Prior to separations, aliquots of cell cultures were mixed 1:1 by volume with ethyl acetate and the mixture was sonicated with a microtip-equipped Branson Sonifier 250 for 3 minutes. Complete cell disruption was confirmed by microscopy. The analysis gave total culture toxin, which was deemed appropriate since preliminary experiments showed >90% of culture toxins were intracellular, corroborating previous findings [46], [47]. Collected ethyl acetate fractions were desalted with Milli-Q water and concentrated with a rotovap. Extraction efficiency was determined in every fraction by the addition of an internal standard; efficiency typically ranged from 90–95% [46]. Concentrated fractions were measured for brevetoxins using an Agilent 1100 LC coupled to a Thermo-Finnigan TSQ Quantum triple quadrupole mass spectrometer with an electrospray ion source interface. LC-MS-MS conditions have been previously described in detail [48],[49]. An external standard curve of purified brevetoxins 1, 2, and 3 (World Ocean Solutions, Wilmington, NC, USA) was used to quantify amounts of extracted brevetoxins.

### 2.3 Statistical analysis

**Figure 6:**
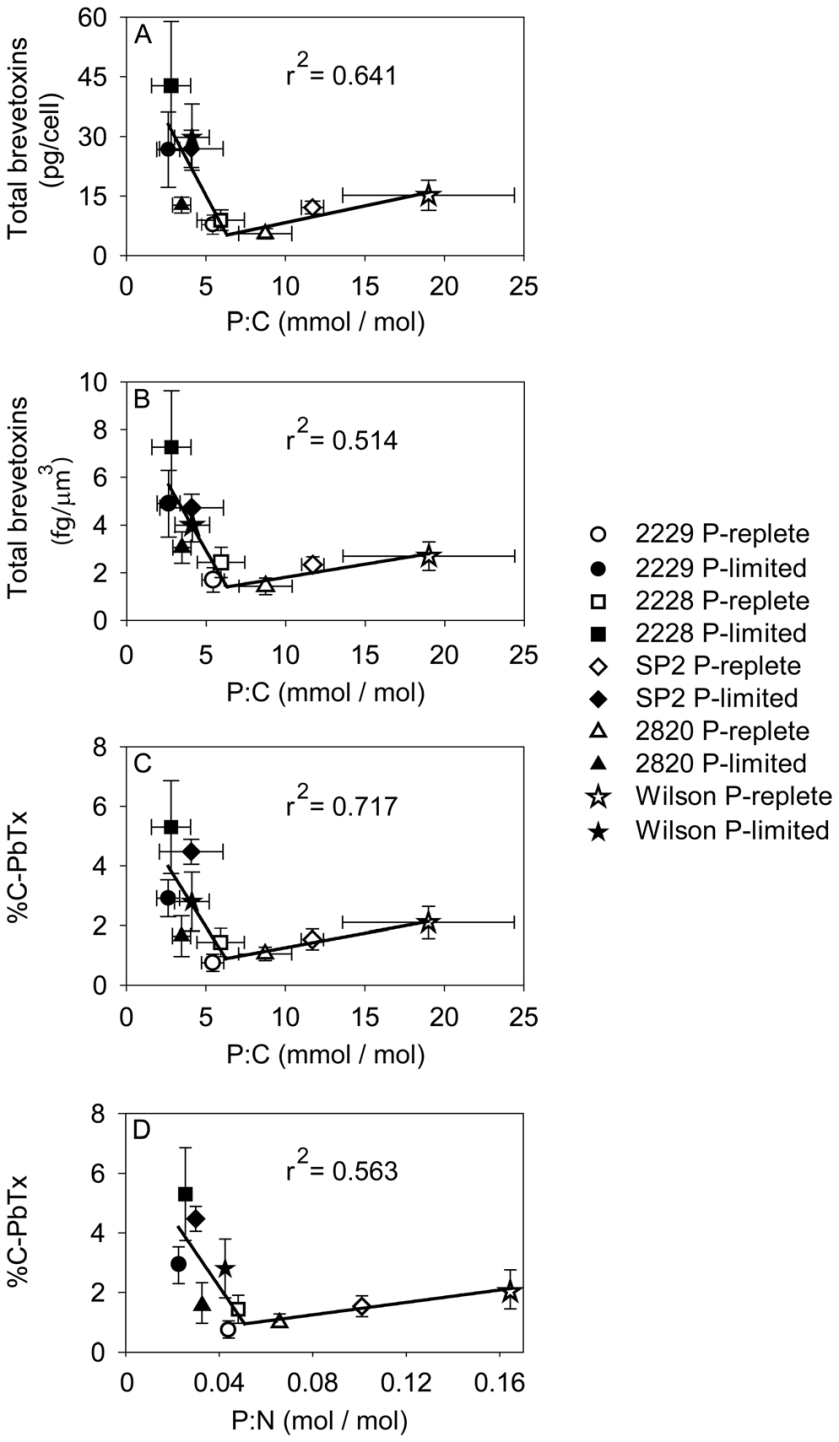
Relationships between cellular brevetoxins and P:C and P:N ratios. (A) Total brevetoxin per cell (pg/cell) vs. P:C ratio (mmol/mol), (B) Total brevetoxins normalized to cell volume (fg/µm^3^) vs. P:C (mmol/mol), (C) Percent of total cell carbon present as brevetoxins (%C-PbTx) vs. P:C (mmol/mol), and (D) Percent of total cell carbon present as brevetoxins (%C-PbTx) vs. P:N (mol/mol). Error bars represent standard deviations of triplicate measurements for both x and y values, except those normalized to carbon, where the cellular C was measured only in duplicate. Data from P-replete and P-limited cultures are indicated by open and filled symbols, respectively. Two segment piecewise linear regressions were fit to the data in each panel using the SigmaPlot® 11.2 graphical analysis program.

**Figure 7:**
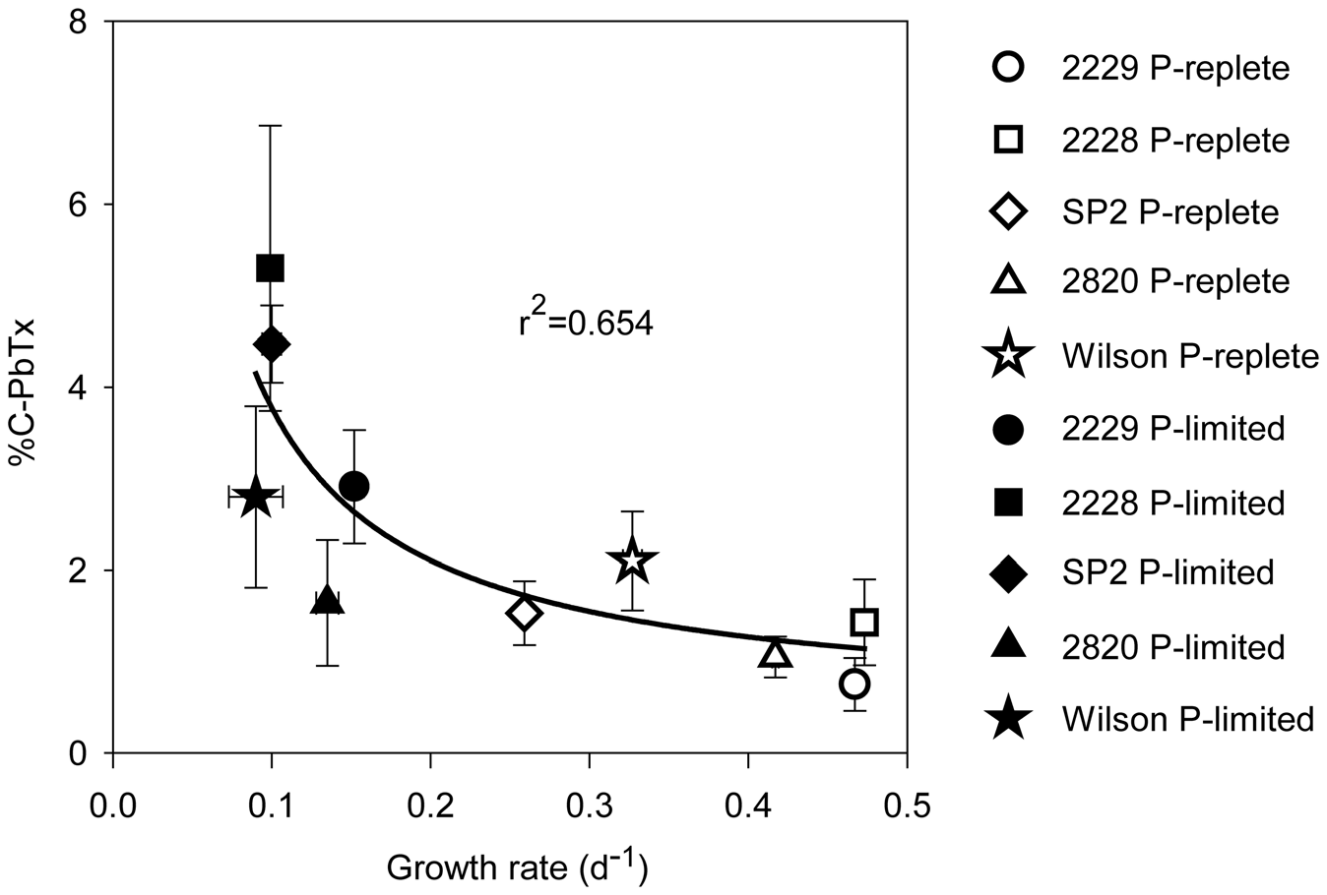
Relationship between cellular brevetoxins expressed as a percent of cell carbon (%C-PbTx) and specific growth rate (d^-1^). Results from P-replete and P-limited cultures are indicated by open and filled symbols, respectively. Error bars indicate standard deviations of triplicate measurements of brevetoxins and duplicate measurements of cell carbon. The best fit curve for the data was obtained using the iterative SigmaPlot® 11.2 graphical analysis program.

**Figure 8:**
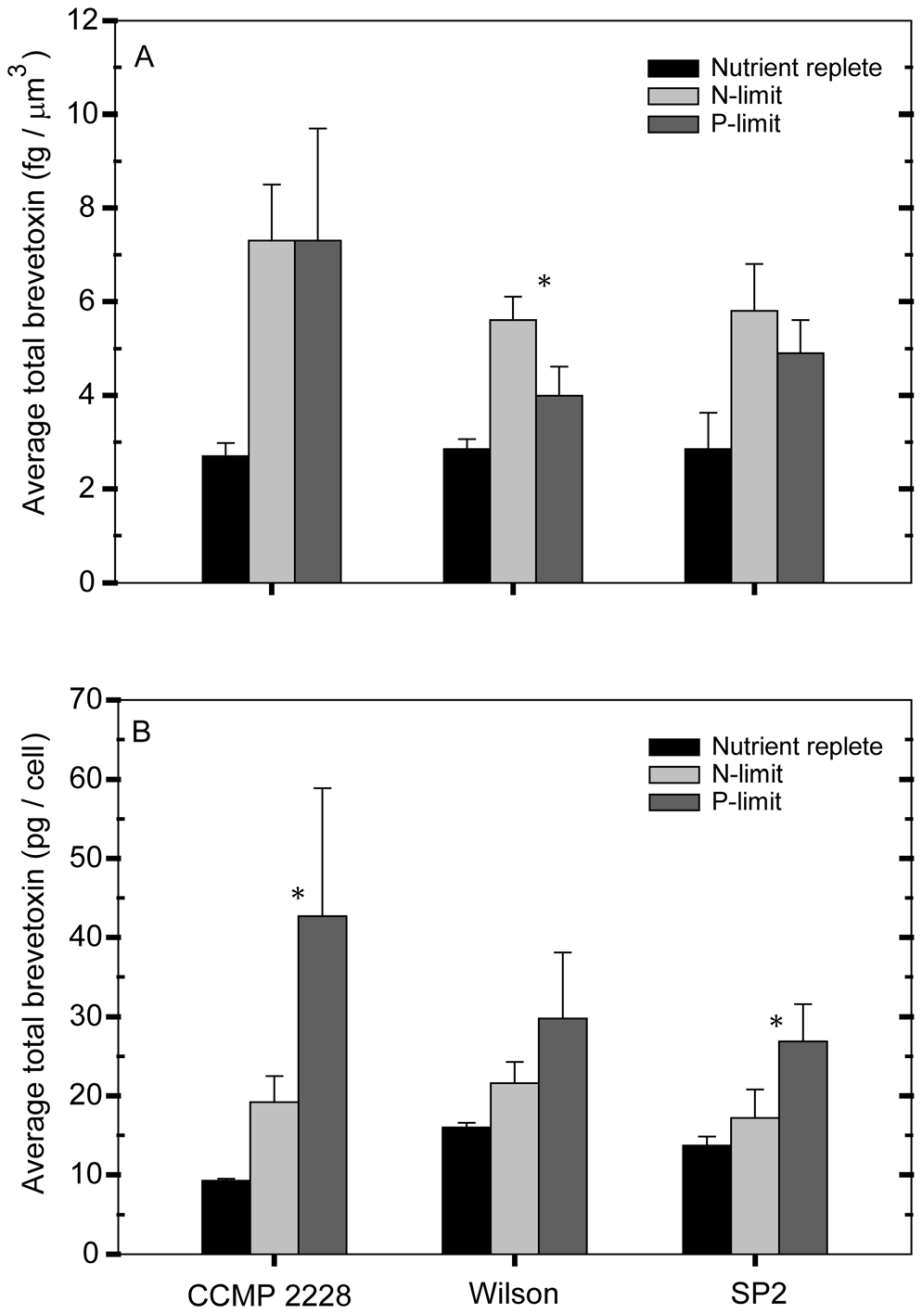
Comparison of N- and P-limitation studies of *Karenia brevis*. Average total brevetoxin normalized (A) to cell volume (fg/µm^3^), and (B) to average total brevetoxin per cell (pg/cell) in nutrient-replete and P-limited cultures in the present study and nutrient-replete and N-limited cultures in a previous study [20]. Average N- and P-limitation values were calculated using data measured after the onset of growth limitation (see methods). Data are presented for the three *Karenia brevis* strains common to both studies. Statistical differences were assessed using a Kruskal-Wallis one-way ANOVA (p<0.05). All P-limitation treatments were significantly different from the control values. All of the N-limited treatments, with the exception of the Wilson and SP2 strains in panel B, were significantly different from the controls. An asterisk (_*_) denotes when the N- and P-limitation toxin values for a particular strain were significantly different.

Mean values were computed for cell toxin and cell composition data (see Table 3 and Figs. 6, 7, 8). All mean values for P-limited semi-continuous batch cultures are based on data collected after the growth of *Karenia brevis* had slowed from P-limitation, which initially occurred on day 9 for strain CCMP 2229, day 10 for the Wilson strain, and day 13 for strains CCMP 2228, SP2 and CCMP 2820. Mean values for nutrient-replete control cultures growing at their maximum rates were collected throughout each experiment. All errors reported in thes and tables represent the standard deviation of triplicate measurements, except for cellular nitrogen and carbon, where only duplicate measurements were made. The number of sampling points used to calculate each mean and standard deviation varied between 6 and 8. The relationships between brevetoxins (normalized per cell, per unit cell volume, or as a percent of cellular carbon [%C-PbTx]) versus cellular P:C or P:N ratios, exhibited positive slopes in the nutrient replete treatments where the ratios were higher. In contrast, these same relationships exhibited a negative slope under P-limited growth where P:C and P:N ratios were lower (Fig. 6A–D). To account for these differing slopes, the data were fitted to two segment piecewise linear regression models using SigmaPlot® 11.2 graph analysis software. The relationship between growth rate and %C-PbTx failed to exhibit a similar biphasic behavior and instead showed continuous negative slopes. This relationship was fitted to a first order polynomial equation using SigmaPlot 11.2 graphical analysis software (Fig. 7).

The data for average total brevetoxins, normalized per cell and per unit biovolume, for three *K. brevis* strains (CCMP 2228, Wilson, and SP2) grown under nutrient-replete, N-limited, and P-limited growth conditions were analyzed for statistical differences using the SigmaPlot® Kruskal-Wallis one-way ANOVA (Fig. 8). The N-limited data were obtained from Hardison et al. [20]. A decrease in growth rate was used in the present and previous study to determine when the cells were P- or N-limited. The nutrient-replete data from the current study and Hardison et al. [20] were combined in this analysis. The number of data points used to calculate each mean and standard deviation varied from 6 to 8 for P- and N-limited cultures and from 6 to 15 for the controls.

## Results

### 3.1 Growth rates

Nutrient replete maximum growth rates among the five *Karenia* strains varied from 0.26 d^−1^ to 0.47 d^−1^, with CCMP 2228 exhibiting the highest rate and strain SP2 the lowest (Table 3). The low-phosphate cultures had approximately the same specific growth rates as the high-P ones for the first ∼8–9 days, after which their growth was limited by phosphate (Figs. 1A, 2A, 3A, 4A, 5A). These P-limited cultures were then resupplied with fresh low-P medium at an average dilution rate of 0.1 d^−1^ beginning on days 10–13, depending on the strain (Figs. 1A, 2A, 3A, 4A, 5A). Measured specific growth rates of the P-limited, semi-continuous cultures ranged from 0.09 d^−1^ for the Wilson strain to 0.15 d^−1^ for strain CCMP 2229 (Table 3). P-limited growth rates for all four strains were 2.6- to 4.8-fold lower than rates in the nutrient-sufficient cultures (Table 3; Figs. 1B, 2B, 3B, 4B, 5B).

### 3.2 Cell density and volume per cell

The cultures were maintained at low total cell volumes (≤ 20 µL_cells_/L_culture_) to minimize decreases in CO_2_ concentrations and attendant pH increases and changes in trace metal nutrient availability [41]. Average volume per cell for nutrient replete cultures ranged from 3,650 µm^3^/cell for strain CCMP 2228 to 5,668 µm^3^/cell for the Wilson strain. These cell volumes remained relatively constant throughout the experiments (Table 3; Figs. 1D, 2D, 3D, 4D, 5D). The mean volume per cell in the *K. brevis* strains increased with the onset of P-limitation of growth rate (Table 3; Figs. 1D, 2D, 3D, 4D, 5D), as is typically observed with growth limitation by phosphate [50]. These increases in mean volume per cell ranged from 6% for strains SP2 and CCMP 2820 to 64% for strain CCMP 2228 (Table 3). The cell volume increase for the latter strain continued for the duration of the experiment, but the other four strains exhibited oscillations in cell volume following the onset of P-limitation (Figs. 1D, 2D, 3D, 4D, 5D). Although some of the volume increases were quite variable, all strains exhibited significant differences in volume per cell between P-replete and P-limited treatments (one-way ANOVA: *p*<0.001 for strains CCMP 2228, CCMP 2229, CCMP 2820 and Wilson; *p*  =  0.03 for strain SP2).

### 3.3 Cellular C, P, and N

Cellular P:C ratios decreased by 1.8- to 3.8-fold with decreasing growth rate in the low-P cultures, indicating that these cultures were P-limited (Table 3; Figs. 1C, 2C, 3C, 4C, 5C). Average cellular P:C ratios for P-replete strains ranged from 6.9±0.9 mmol/mol for strain CCMP 2229 to 20.0±7.5 mmol/mol for the Wilson strain (Table 3). Average P:C ratios were lower in P-limited cells and ranged from 3.5±1.8 mmol/mol for strain CCMP 2228 to 6.2±2.0 mmol/mol for strain CCMP 2820 (Table 3). Mean cellular N:C ratios also decreased under P-limitation in four of the five *K. brevis* strains (all but Wilson), however, the extent of this decrease (1.11- to 1.67-fold) was less than observed for P:C ratios (Table 3).

### 3.4 Chlorophyll *a*

P-replete cultures of the five *K. brevis* strains exhibited mean cellular chl *a* of 1.1 to 1.6 fg/µm^3^ normalized to cell volume (Table 3). Cellular chl *a* decreased with the onset of P-limitation of growth rate and remained lower than P-replete values throughout the experiments (Figs. 1F, 2F, 3F, 4F, 5F). Average decreases ranged from 44% to 68% when expressed on a cell volume basis (Table 3).

### 3.5 Brevetoxins

The dominant brevetoxins (PbTxs) detected in four of the strains were PbTx-1, -2, and -3. The only exception was the Wilson strain which only contained measurable levels of PbTx-2 and PbTx-3. In this study, reported total brevetoxins equal the sum of these three dominant congeners. For each of the strains tested, PbTx-2 was the most abundant brevetoxin, followed by PbTx-1 (when present) and trace amounts of PbTx-3 (< 1 pg/cell). PbTx-1 and PbTx-2 are intracellular congeners whereas PbTx-3 is primarily an extracellular breakdown product of PbTx-2 [51]. The relative distribution of the individual toxin congeners was similar in P-limited and P-replete cultures (data not shown).

Phosphate-replete cells growing at their maximum rates in the high-P media and in the low-P media at the beginning of the time course experiments exhibited similar total brevetoxin concentrations (Figs. 1E,G,H, 2E,G,H, 3E,G,H, 4E,G,H, 5E,G,H). The percent of cell carbon present as brevetoxins (referred to hereafter as %C-PbTx) varied among the nutrient-sufficient cultures and was highest (2.1 and 1.5%, respectively) for the Wilson and SP2 strains and lowest (0.75%) for strain CCMP 2229 (Table 3). As the initially P-replete cells in the low-P media transitioned into P-limitation of growth at approximately day 10, their brevetoxin levels increased on a cell volume (fg/µm^3^), per cell (pg/cell), and per cell carbon basis (%C-PbTx). The pattern of increase, however, was often complex, and depended on both the strain and how a given cellular attribute was normalized (i.e., per cell, cell volume, or cell C; Figs. 1E,G,H, 2E,G,H, 3E,G,H, 4E,G,H, 5E,G,H). All but one of the strains (SP2) showed peaks in brevetoxin values at varying times after the onset of P-limitation, followed by decreasing values thereafter. For strain SP2, the cellular toxin per cell and per unit of cell volume continued to increase from the onset of P-limitation after day 7 until the end of the experiment on day 29, while the %C-PbTx remained nearly constant after day 12 (Fig. 3E,G,H). Peak values for total brevetoxin per cell for the strains examined were 65 pg/cell for strain CCMP 2228, 43 pg/cell for the Wilson strain, 36 pg/cell for SP2, 16 pg/cell for CCMP 2820, and 48 pg/cell for CCMP 2229 (Figs. 1E, 2E, 3E, 4E, 5E). These peak values were 2.9- to 7.3-fold higher than the mean values observed in nutrient-replete cells, with strains CCMP 2228 and CCMP 2229 showing the highest increases. A similar trend was observed when toxins were normalized to biovolume and cell carbon (Figs. 1G,H, 2G,H, 3G,H, 4G,H, 5G,H). Brevetoxins normalized to cell volume exhibited maximum increases of 1.9- to 4.6-fold under P-limitation relative to nutrient sufficient values and %C-PbTx exhibited maximum increases of 2.3- to 4.9-fold. As with toxin values per cell, brevetoxins per unit of cell volume for strains CCMP 2228 and CCMP 2229 showed the largest increase in maximum values (4.6-fold). Strains SP2 and CCMP 2820 had intermediate increases (2.7- and 2.8-fold), and the Wilson strain had the least increase (1.9-fold). Maximum increases in brevetoxins normalized to cell C (%C-PbTx) were also largest for strains CCMP 2229 and 2228 (4.9- and 4.8-fold), intermediate for strains SP2 and CCMP 2820 (3.4- and 3.1-fold), and smallest for the Wilson strain (2.3-fold). A noticeable difference in the magnitude of the cellular brevetoxin increase is observed depending on which normalization factor is used. On a per biovolume or cell C basis, brevetoxin increases were not as substantial as when toxins were normalized per cell. This behavior reflects the fact that the P-limited cells increased in size and thus had higher volumes and carbon mass per cell. This size increase meant that a given increase in toxin per unit cell volume or carbon would automatically translate to an even larger increase in toxin per cell. Regardless of how cellular brevetoxins were normalized, the toxins in P-limited cultures remained elevated relative to nutrient replete cultures for the duration of the experiments. Nutrient replete controls maintained similar low levels of cellular brevetoxins throughout the experiments (Figs. 1E,G,H, 2E,G,H, 3E,G,H, 4E,G,H, 5E,G,H).

Mean cellular brevetoxins normalized per cell, cell volume, and cell C showed consistent relationships with the mean cellular P:C ratio among all strains under both nutrient sufficiency and P-limitation. Cellular brevetoxins were positively correlated with P:C values in P-sufficient cells (P:C ∼ 6 to 20 mmol/mol; r^2^  =  0.75 for linear regression including only the P-sufficient data). This positive correlation was associated with the fact that different isolates (particularly SP2 and Wilson) exhibited inherently different P:C ratios as well as different levels of carbon investment in brevetoxins when growing maximally under nutrient-replete conditions. Under nutrient-replete growth conditions, the strains with inherently slower growth rates (Wilson and SP2) had up to three-fold higher cellular P:C ratios than the slowest growing strains (CCMP 2228 and CCMP 2229), apparently due to “luxury consumption” of phosphate in excess of the minimum amount needed to support growth (Table 3). The same slower growing strains also showed a greater carbon investment in PbTxs (Table 3; Figs. 6, 7). By contrast, at low P:C ratios (<6) associated with P-limitation of growth rate, the opposite behavior occurred, and decreases in P:C values were accompanied by a sharp rise in cellular brevetoxins (Fig. 6A–C). This same two-phase relationship was observed between P:N and %C-PbTx (Fig. 6D). These relationships were analyzed by two segment piecewise linear regressions. The r^2^ values for these regressions varied between 0.51 and 0.72 (Fig. 6A–D). In contrast, a monotonically decreasing relationship was observed between cellular brevetoxins as a percent of cellular C (%C-PbTx) and specific growth rate (r^2^  =  0.65, *p*<0.005; Fig. 7). Thus, as growth rates decrease, there is an increased investment of cellular carbon in brevetoxins irrespective of whether the growth rate change was related to inter-strain variations in maximum growth rate or to decreases in growth rate among the strains caused by phosphate deficiency (Fig. 7).

### 3.6 Comparison of N- and P-limitation

Figure 8 shows a comparison of average brevetoxin values under nutrient replete, N-limited, and P-limited conditions based on data from strains CCMP 2228, Wilson, and SP2 in the present study and an N-limitation study we previously conducted [20]. Results vary depending on how the toxin values are normalized. Both N- and P-limitation increased cellular brevetoxins per unit of cell volume in all strains tested, but to varying degrees. Strain CCMP 2228 exhibited the same increase in toxins per unit of cell volume under N- and P-limitation, but the Wilson and SP2 strains showed a slightly higher increase under N-limitation, which was statistically different only for the Wilson strain (*p*<0.05). N-limitation caused an average 29% decrease in volume per cell in the three strains [20] while P-limitation caused an average 35% increase (Table 3). Because of these opposing changes in cell size, the P-limited cells on average had a 2.0-fold higher volume per cell, and consequently, had higher values of brevetoxin per cell than the N-limited cells. These differences were statistically significant for all but the Wilson strain (*p*<0.05) (Fig. 8B).

## Discussion

### 4.1 Evolutionary tradeoffs between grazing defense and growth

*Karenia brevis* forms large ecosystem disruptive algal blooms in the Gulf of Mexico that adversely affect human and animal health [33], [52], [53]. The role of nutrients in initiating and maintaining blooms, and in controlling cellular toxicity remains controversial [15], [18], [20]. This study was undertaken to examine how phosphate limitation regulates the growth and carbon investment in brevetoxins in different *Karenia brevis* strains. P-limitation is of interest because evidence indicates that regions where *Karenia* blooms occur can become transiently limited by phosphate [26], [54].

Evolutionary theory predicts that as microalgal growth slows in response to nutrient limitation, cells will apportion a greater percentage of their fixed carbon into defense mechanisms [36]. This hypothesis is often referred to as the carbon:nutrient balance (CNB) hypothesis. It was originally based on observations from vascular plants, which were shown to divert a greater portion of their fixed carbon from growth to defense under resource limitation [35], [55]. Plant and algal defenses often involve the production of anti-grazing compounds (e.g., toxins) as well as the formation of thicker cell walls, spines, or other structures [37], [56]. In *Karenia brevis* brevetoxins (PbTxs) have been shown to be important anti-grazing compounds [10], [57]. These potent voltage-sensitive sodium channel activators disrupt nerve function [58], which accounts for why *Karenia brevis* blooms cause neurotoxic shellfish poisoning, massive fish kills, marine mammal and bird mortalities, and the formation of toxic aerosols along affected beaches [3], [4], [59], [60]. In the present study average PbTx per unit of cell volume increased 1.5- to 2.9-fold from P-replete to P-limited growth conditions and similar increases were observed under N-limitation in a previous study [20]. These findings are consistent with the CNB hypothesis and indicate that P- and N-limited blooms are more likely to create greater adverse ecosystem and human health effects than those where algal growth is not limited by nutrients.

The data also indicated that some strains were more toxic than others (Figs. 6A–D; 7). Under nutrient-replete growth conditions, the investments of cell C in PbTxs varied between 0.75 and 2% of total cellular C. The corresponding range of mean %C-PbTx for P-limited cells was from ∼1.5 to 5.3% (Table 3), with maximum values ranging from 3 to 6% (Figs. 1H, 2H, 3H, 4H, 5H). These latter values represent a significant carbon investment in anti-grazing defenses as growth slowed. Interestingly, although all strains increased their investment in PbTxs under P-limitation, some diverted more of the total carbon pool to PbTxs than others (Fig. 6A–D). The factors governing the differences in this investment are not immediately obvious until %C-PbTx is plotted against growth rate (Fig. 7). That plot showed that growth rate and %C-PbTx were inversely related for both P-replete and P-limited cells (*p*<0.005; Fig. 7). Thus, the slower the growth rate under all conditions (P-replete and P-limited) the greater the cellular investment in PbTxs, known grazing defense toxins [10], [57]. These data are consistent with evolutionary tradeoffs between C invested in supporting growth and reproduction and that invested in the production of PbTxs and other grazing defenses [37], [61]. It is likely that if we assessed more strains or varying degrees of P-limitation of growth rate, there would be a continual gradation in %C-PbTx vs. growth rate. Because net algal population growth is largely dependent on rates of growth and reproduction minus rates of grazing mortality losses, this variation would be selectively advantageous at the population level in aquatic environments where grazing pressures and growth limiting nutrients continually fluctuate on different temporal and spatial scales (Fig. 7) [10], [57]. This trade-off linked variability among strains promotes genetic diversity within populations of algal species, which in turn permits the adaptation of populations to changing conditions. Similar evolutionary tradeoffs have been observed in terrestrial plants [56] and in other phytoplankton [61]. For example, a study of nutrient acquisition in 13 algal strains representing 11 separate species, the species or strains with lower nutrient uptake rates and growth rates for their size were also poorly grazed or assimilated, suggesting higher levels of grazing defenses [61]. These results are consistent with the growth and defense relationship exhibited by the different *K. brevis* strains under nutrient-sufficient and P-limited growth conditions, where the least amount of carbon was associated with brevetoxins in strains growing at the fastest rates, and more carbon was allocated to these defensive compounds under reduced growth rates (Fig. 7). Furthermore, the C contained in PbTxs likely represents only a portion of the total C expended for cellular defenses. Additional C (and N) would be needed for the production of PbTx biosynthetic enzymes and for the synthesis of unidentified toxic compounds produced by *K. brevis*, that inhibit the growth of other algae [62]. Similar production of multiple defensive compounds is widespread in terrestrial plants [56].

These results also raise an interesting question of whether the increased PbTx to cell C ratios under P-limited growth represents a net up-regulation of cellular toxin production rates. To answer this question we note that during steady state growth, the cellular toxin:C ratio equals the C-normalized production rate divided by the specific growth rate, the effective rate of biodilution. Because of this relationship, an increase- in cellular toxins can be caused by an increase in production rates, a decrease in growth rate, or by changes in both. Computed steady-state PbTx production rates in the P-limited cells were generally the same or lower than those in the faster growing nutrient-replete cells (Table 3). A possible exception was strain CCMP 2229, where there was a 26% increase in the production rate under P-limitation, but the increase was not statistically significant (*p*  =  0.15, t-test). The results indicate that the increased toxin to C ratios in the P-limited cells are achieved by down-regulating C investment in biomolecules involved in C-fixation and growth, while maintaining the same PbTx production rate or down-regulating the rate of PbTx production to a lesser degree than that for other cellular constituents. For example, the average 45±10% decrease in the cellular chl *a* concentration and 21±16% decrease in the cellular N:C ratio was likely due to a down-regulation of the net synthesis of pigments and proteins involved in photosynthesis and other cellular processes needed to support growth (Table 3). Thus, the cells preferentially maintain production of PbTxs over other cellular constituents when growth slows leading to an increase in cellular toxin concentrations.

The CNB hypothesis also predicts that the investment in PbTxs will be highest during the period when growth rate initially slows from the onset of nutrient limitation. During this time the growth slows more quickly than the photosynthetic apparatus can be down-regulated, which results in a temporary imbalance between photosynthetic C-fixation and the cellular C demand for growth (Figs. 1F, 2F, 3F, 4F, 5F). To avoid over-reduction of the photosynthetic apparatus, and consequent production of toxic reactive oxygen species [63], the CNB hypothesis states that cells divert some of the “excess” fixed carbon into defensive compounds. In this study, the %C-PbTx rapidly increased in all five of the strains during this unbalanced growth period, but then subsequently declined to different degrees in all strains except SP2 once photosynthetic C-fixation and growth began to come back into balance (Figs. 1H, 2H, 3H, 4H, 5H). The same trends were observed when the data were expressed as PbTx per cell or per unit of cell volume (Figs. 1E,G–5E,G). The timing and magnitude of the maxima, however, were not always the same as for %C-PbTx because of concomitant changes in volume per cell (Figs. 1D, 2D, 3D, 4D, 5D) and cell C:volume ratios (Table 3). The transient peaks in PbTx per cell in the P-limited cells were 16–65 pg/cell, depending on the strain, with the higher of these values corresponding to the upper values observed in the field during blooms (Table 1). This observation suggests that blooms will reach their maximum toxicity in the early phases of nutrient limitation.

As P-limitation progresses past early transient phases into the later phases of growth limitation, toxin levels began to stabilize as the cells reached an acclimated (relatively constant) P-limited growth rate. Interestingly at this point, when cells no longer need to “dump” excess fixed carbon, %C-PbTx values remain significantly higher than observed in the P-replete cells, but the degree to which this occurs varies among strains. As noted previously, these data indicate that even after down regulation of chl *a* brings C-fixation and growth back into balance, *K. brevis* cells continue to invest a greater portion of their total cellular C in PbTxs, apparently to lower grazer-linked mortality rates when P-limitation significantly reduces cellular rates of growth and reproduction.

The data obtained in this paper provides an unusual opportunity to examine how well P:N and P:C ratios predict cellular toxicity, as these ratios have been typically used in past to demonstrate relationships between toxin (or toxicity) per cell and N- and P-limitation [34]. For example, culture studies with *Alexandrium tamarense*, *A. minutum*, *Prymnesium parvum*, and *Chrysochromulina polylepis* have consistently shown that as the cellular P:N ratio decreases below ∼0.0625 (i.e., the N:P increases above 16, the Redfield ratio [64]) under P-limitation of growth rate, toxin per cell increased steadily as long as P is not an essential component of the toxin being synthesized [34]. However, relationships between cellular toxins and specific growth rate were not presented. By comparison, our results indicate that P:N and P:C ratios are less accurate predictors of toxicity than growth rate because different ratios correspond with the same cellular PbTx concentrations, whether these are normalized on a per cell, cell volume, or cell carbon basis (Figs. 6, 7). These equivalent toxin concentrations at different elemental ratios was a result of inter-strain variations in how toxin levels and P:C and P:N ratios responded to P-limited and P-sufficient growth conditions. Under P-limitation, growth rates slowed, P:C and P:N ratios declined, and cellular brevetoxins increased. This resulted in a negative relationship between cellular toxins and P:C and P:N ratios. By contrast, in the P-sufficient treatments, the strains with the lowest maximum growth rates exhibited higher toxin contents and elemental P ratios than the faster growing strains (Fig. 6). As a result, cellular PbTx concentrations were positively correlated with P:C and P:N ratios in the P-replete treatments.

The high cellular N:C and N:P ratios in the slower growing P-replete strains, likely represents luxury uptake of phosphate in excess of that needed to support growth, which is well known to occur in phytoplankton [65], [66]. These slower growing strains would presumably be better adapted to nutrient limiting conditions, which is also consistent with their higher basal cellular concentrations of PbTxs and associated grazing defenses [61]. The higher luxury consumption in these strains would also provide them with the necessary phosphate to support growth once external phosphate concentrations become depleted at high cell densities.

The combined data from this study clearly show that growth rate is the best predictor of cellular toxicity. However, P:C and P:N ratios may be more useful in assessing nutrient limitation in the field because of the difficulty in quantifying specific growth rates in natural *Karenia* populations.

Although *Karenia brevis* responds to P- and N-limitation by increasing all brevetoxin congeners (PbTx-1, -2, and -3) to the same extent yielding no change in congener ratios, this does not occur in all toxic algae. The dinoflagellate *Karlodinium veneficum* exhibits a different strategy, and increases its toxicity by preferentially increasing the cellular concentration of the more potent karlotoxin-1 (KmTx-1) relative to that of the less toxic KmTx-2 [32].

### 4.2 Brevetoxin levels in cultures versus those observed in the field

A survey of field and laboratory measurements shows a cellular brevetoxin range of 1–68 pg/cell (Table 1). Our previous study of N-limitation effects indicated a range of PbTx values in nutrient-sufficient and N-limited *K. brevis* of only 7–25 pg/cell [20]. By contrast, the values in the present study ranged from 5–65 pg/cell (Figs. 1E, 2E, 3E, 4E, 5E). This larger range in toxicity per cell was due to both inherent strain differences and to higher PbTx/cell under P-limitation of growth rate. PbTx per cell in the five *K. brevis* strains investigated ranged from 5–15 pg/cell under nutrient sufficiency (Table 3) and increased by up to 2.9- to 7.3-fold under P-limitation (Figs. 1E, 2E, 3E, 4E, 5E). These data indicate that P-limitation, rather than N-limitation can account for the full upper range of PbTx per cell observed in the field. They are also consistent with transient P-limitation having occurred during the time when some of the cells were collected in the field [67].

The influence of P-limitation on toxin per cell values in the field is only relevant if bloom waters in the Gulf of Mexico are indeed P-limited, either on a continuing or intermittent basis. Generally, the world’s ocean waters are considered to be N-limited except in the few regions where the ratio of net N- to P-inputs from various sources exceeds the average value in nutrient sufficient phytoplankton (generally 16:1) [68], [69]. In the Gulf of Mexico there appears to be a number of factors operating simultaneously, which may be fueling a long-term transition from a predominantly N-limited to a P-limited system. These factors include increases in atmospheric dust deposition which supplies iron and promotes increased N-fixation by cyanobacteria, N-enriched river inputs, and atmospheric deposition of bioactive N species. In the Gulf of Mexico, all these factors are shifting the N:P stoichiometry towards P-limitation of algal growth rate.

An increasing body of evidence indicates that iron limitation of N_2_-fixation by cyanobacteria is a major contributor to the N-limited state of most surface ocean waters [70]–[72] However, in the tropical and subtropical North Atlantic and adjacent oligotrophic waters of the Gulf of Mexico and West Florida Shelf, atmospheric deposition of iron from Saharan dust promotes N_2_-fixation by the cyanobacterium *Trichodesmium*, thereby driving these waters towards P-limitation of algal growth [70], [73]. *Trichodesmium* has both behavioral and biochemical mechanisms that allow effective uptake of inorganic and organic phosphorus, even when present at extremely low concentrations [74]. In oligotrophic waters, N_2_-fixation and concomitant depletion of P during *Trichodesmium* blooms may intensify P-limitation in co-occurring *Karenia brevis* populations [18], [75]. Long term climate monitoring has shown that atmospheric deposition of Saharan dust to the ocean has increased 3- to 4-fold since the 1960’s as a result of drought conditions in the sub-Saharan region [76]. If these trends continue, enhanced N_2_-fixation should accelerate the shift from N- to P-limited phytoplankton growth currently occurring in the subtropical North Atlantic and Gulf of Mexico.

Another important source of nutrients to the Gulf of Mexico (GOM) is the Mississippi River. Shifts in agricultural practices and regulation of phosphate inputs over the past 50 years have led to both a significant increase in nutrient inputs to the GOM [77], [78] and a fundamental shift toward higher N:P ratios, thereby favoring P-limitation of algal growth. Specifically, N:P ratios increased from 9:1 in 1966 to 38:1 in 1996, and to 20-60:1 in 2003-2008 [23], [79], [80]. Long term data from the Mississippi outflow in the GOM shows that N:P loading ratios rose from 16 in 1960 to 24 in 1987 [23]. Currently, the Mississippi River is considered to be P-limited [23], [79]. This assessment was confirmed by seasonal bioassays in the Mississippi plume in the Gulf of Mexico in 2001, where >75% of the stations surveyed in the summer and early fall were found to be P-limited [25]. Although the outflow of the Mississippi advects towards Texas from winter to spring, these nutrient-rich waters are transported towards the West Florida Shelf in summer and fall as a result of a shift in prevailing winds and may represent a significant nutrient source to *Karenia* blooms [15], [81].

Increasing atmospheric inputs of reactive nitrogen from anthropogenic sources, such as NO_x_ inputs from fossil fuel burning, may also contribute to a shift from N- to P-limitation of algal growth in the Gulf of Mexico. Atmospheric deposition from anthropogenic sources has become a major contributor of biologically available fixed nitrogen to the oceans and is projected to increase 4-fold by 2030. It may surpass N_2_-fixation as the primary source of bioactive N for the world’s oceans in the next 50 years [24]. In contrast, atmospheric deposition of P is relatively small [82], [83].

A recent study of nutrient inputs and ratios on the West Florida Shelf provides evidence for how frequently P-limitation is likely to occur in the different regions along the shelf [26]. Based on particulate N:P ratios, Heil et al. [26] concluded that the northern areas of the West Florida Shelf were N-limited and that P-limitation became increasingly more common toward the western Florida Bay and Florida Keys. They proposed that the observed gradient was partly due to P inputs from the drainage of phosphate mines east of Tampa Bay resulting in N-limitation on the northern shelf. It was further hypothesized that the greater P-limitation in the south was caused by the outflow of waters from the Everglades with high N:P ratios, and the presence of carbonate sediments in Florida Bay and the southwest Florida shelf that bind and sequester phosphate from the overlying waters [54]. Therefore, *K. brevis* blooms may become P-limited as they advect with southward flowing coastal currents along the West Florida Shelf.

### 4.3 Ecosystem effects and management implications

The 2- to 5-fold increase in PbTxs per mole cell carbon or unit of cell volume during periods of transient P-limitation in this study and N-limitation in a previous study [20] means that marine ecosystems are likely exposed to significantly different toxin levels depending on the nutrient status of the cells. Brevetoxins are largely contained intracellularly during the early stages of bloom development; however, as blooms age and incur apoptosis and cell lysis, the toxins are released into the surrounding waters [20]. Therefore, elevated cellular brevetoxin levels caused by P-limitation may result in higher inputs of brevetoxins into the environment. Because the released PbTxs absorb onto biological surfaces such as sea grass fronds and accumulate in consuming organisms, high toxin levels may persist in the food chain long after a bloom has subsided [84], [85].

Higher cellular toxin levels can also disrupt grazer-prey interactions. Copepod grazing on *K. brevis* has been shown to decrease as PbTx per cell increases [10], [57]. The resultant lower grazing mortality rates should provide *K. brevis* with a selective advantage over competitors. In addition, as grazing on *Karenia* is reduced or eliminated, grazer-mediated nutrient recycling is diminished and the lower recycling increases nutrient limitation of *Karenia brevis* growth rates, ultimately yielding even more toxic cells. This sets up a positive feedback interaction with increasing *K. brevis* cell densities having progressively greater negative impacts on the grazing, nutrient recycling and nutrient availability. This dynamic may foster the development and persistence of *Karenia brevis* and other ecosystem disruptive algal bloom (EDAB) species [33], [86].

The data presented here and previously [20] indicate that brevetoxins increase under both N- and P-limitation. The increases in brevetoxins per cell under P-limitation, however, are roughly twice those under N-limitation due to differential changes in cell size. Thus, for the same concentration of cells, a P-limited bloom would be twice as toxic as an N-limited one for the same degree of growth rate limitation. This difference is of significance since the severity of a bloom for the management of shellfish bed closures is based upon *Karenia brevis* cell concentrations, with the false assumption that brevetoxin concentrations per cell do not vary [2]. Therefore, public safety could be compromised if a bloom became nutrient-limited, and the risk could be much higher if the bloom was P-limited rather than N-limited. The potential for N- and P-limitation to increase bloom toxicity provides further support for the need to directly measure brevetoxins as well as cell numbers to accurately assess the potential impact of *Karenia brevis* blooms on human health and to enable proper regulation of shellfish bed closures by coastal managers [87].
